# Lumbosacral transitional vertebra in spondylolisthesis: frequency, demographic findings, and clinical characteristics

**DOI:** 10.1186/s12891-024-07318-z

**Published:** 2024-03-27

**Authors:** Mehdi Mahmoodkhani, Arvin Naeimi, Amirhossein Zohrevand, Arian Rabbanifard, Majid Rezvani

**Affiliations:** 1 Department of Neurosurgery, Isfahan University of Medical Sciences, Isfahan, Iran; 2 Student Research Committee, School of Medicine, Guilan University of Medical Sciences, Rasht, Iran; 3 Department of Surgery, School of Medicine, Babol University of Medical Sciences, Babol, Iran; 4 Faculty of Medicine, Isfahan University of Medical Sciences, Isfahan, Iran; 5 Department of Neurosurgery, School of Medicine, Neurosciences Research Center, Al-Zahra Hospital, Isfahan University of Medical Sciences, Isfahan, Iran

**Keywords:** Sacralization, Lumbarization, Spondylolisthesis, Lumbosacral transitional vertebrae, LSTV

## Abstract

**Background:**

The association of LSTV with low back pain has been debated in the literature for nearly a century, but the relationship between LSTV and spondylolisthesis is still under discussion. There is currently no valid information about LSTV’s prevalence in Iran. This study investigated the relationship between the presence of LSTV and lumbosacral spondylolisthesis regarding frequency, gender and age variation, grade and level of spondylolisthesis, and clinical signs and symptoms.

**Methods:**

This cross-sectional study included spondylolisthesis patients admitted for surgery between March 2021 to December 2022. All patients underwent CT imaging. After evaluating medical records, the baseline data were collected. Patients were categorized into No LSTV, Sacralization, and Lumbarization groups. Demographic and clinical characteristics of the studied groups were compared using an independent T-test and Chi-Square. Multiple logistic regression was used to assess the age and sex variations between groups.

**Results:**

219 patients with a mean age of 57.07 ± 11.04 were included. A significant relationship was observed between the presence of sacralization and gender diversity with female predominance (*P* = 0.01). The level of spondylolisthesis and the presence of motor deficits (paresis) significantly differed among study groups (*P* < 0.05). Sacralization group exhibited a greater prevalence of higher grades of listhesis compared to the other groups.

**Conclusions:**

LSTV is frequently seen in spondylolisthesis patients. Sacralization is the common type of LSTV in spondylolisthesis patients, possibly leading to an increased risk for higher grades of vertebral slip and higher rates of motor deficit signs and symptoms. The presence of sacralization results in a significant increase in the incidence of higher levels of spondylolisthesis, especially the L4-L5*(sacralized L5) level. There is no relationship between age and the presence of LSTV in spondylolisthesis.

## Background

Lumbosacral transitional vertebrae (LSTV) are congenital spinal anomalies where an elongated transverse process of the last lumbar vertebra (L5) fuses with the first sacral segment in variable degrees. LSTV, as a morphological variation, ranges from partial/complete sacralization of L5 to partial/complete lumbarization of S1 [1]. In fact, sacralization is a condition where the L5 is at least partially connected directly to the sacral bone. Lumbarization occurs when the first sacral segment (S1) is not completely fused with the second sacral segment (S2). Accordingly, there is an additional articulated vertebra which is anatomically defined as the last lumbar vertebra. In most cases, the transition is incomplete or unilateral [2]. The prevalence of LSTV in the normal population varies among different studies, ranging from 4 to 36% in various reports [3]. A wide range of LSTV prevalence is likely due to differences in individual diagnostic and classification criteria, observer error, imaging techniques, and confounding factors within the studied population [3]. LSTVs are usually asymptomatic without any clinical signs or symptoms and are discovered almost always incidentally by imaging for other purposes. However, for nearly a century, the literature has debated the correlation between LSTV and low back pain [4].

Low back pain (LBP) is one of the most common causes of adult disability. A lumbar segmental instability (LSI) might be one of the causes of LBP. LSI prevalence can be as high as 57% of patients with chronic LBP [5]. The LSI is related to the proper and balanced working of the three subsystems: active, passive, and neural control [6]. The abnormal function of one of them can lead to an overload of others and cause pain and reduced quality of life [7]. LSI symptoms might lead to spondylolisthesis [6, 8]. However, LSI is difficult to define, whether or not spondylolisthesis is involved [9]. Spondylolisthesis recognized as another prevalent source of lower back pain [10], represents a type of segmental instability that includes LSI [9, 10]. Spondylolisthesis can occur by many causes including degeneration, isthmic defect, dysplasia, or trauma [9, 11]. Nevertheless, the “isthmic,” associated with spondylolysis, and “degenerative,” related to degeneration of the posterior facet joints and/or intervertebral disc are considered two major etiologies of spondylolisthesis. Degenerative spondylolisthesis occurs mainly at the L4-L5 level [12, 13] as opposed to isthmic spondylolisthesis, which occurs most often at the lumbosacral level (L5-S1) [14]. Grade I spondylolisthesis accounts for approximately 75% of all cases [15]. The treatment of spondylolisthesis can be conservative or invasive. Despite conservative treatment recommendations, most spondylolisthesis patients will eventually necessitate invasive treatment. Besides, many spondylolisthesis patients are diagnosed when they meet the criteria for invasive treatment. The most common types of surgery used to correct spondylolisthesis are laminectomy and/or fusion (conventional or interbody fusion). In most cases, both procedures are combined, which results in a clinically significant improvement compared to laminectomy alone [16].

Although the association of LSTV with low back pain has been debated in the literature [4, 17], the relationship between LSTV and spondylolisthesis is still under discussion. In addition, there is currently no valid information about LSTV’s prevalence in Iran. Hence, this study investigated the relationship between the presence of LSTV and lumbosacral spondylolisthesis regarding frequency, sex, age, grade, and level of spondylolisthesis and clinical signs and symptoms.

## Materials and methods

### Study design and participants

This cross-sectional study was conducted from March 2021 to December 2022 to evaluate the population of spondylolisthesis patients who were admitted for surgery to Kashani and Alzahra hospitals in Isfahan, Iran. All patients aged 18 to 85 with a diagnosis of spondylolisthesis admitted for surgery were included in the study. This study also encompassed patients with grade 1 spondylolisthesis (according to the Meyerding classification) who, despite lifestyle changes, medication intake, and use of braces, were resistant to conservative medical treatment and were subsequently referred for surgery. Patients with the following criteria were excluded from the (1) incomplete or unreliable medical records; (2) under simultaneous treatment of other diseases besides spondylolisthesis during hospitalization; (3) temporary hospitalization for nerve block interventions during the study period in people diagnosed with spondylolisthesis or treated before March 2021; (4) malignancy with or without chemotherapy; (5) taking an immunosuppressant medications or hormone replacement therapy; (6) history of documented spinal osteomyelitis, spinal discopathy, herniated disc, spinal stenosis or rheumatologic diseases; (7) history of previous CNS surgery or spinal injury due to trauma.; (8) lack of consent to participate or perform CT imaging. The study was approved by the ethics committee of the Isfahan University of Medical Sciences (IR.MUI.MED.REC.1400.101) in accordance with the World Medical Association’s code of ethics (Declaration of Helsinki, revised in Brazil 2013). Written informed consent was obtained from all participants.

### Data collection

**Fig. 1:**
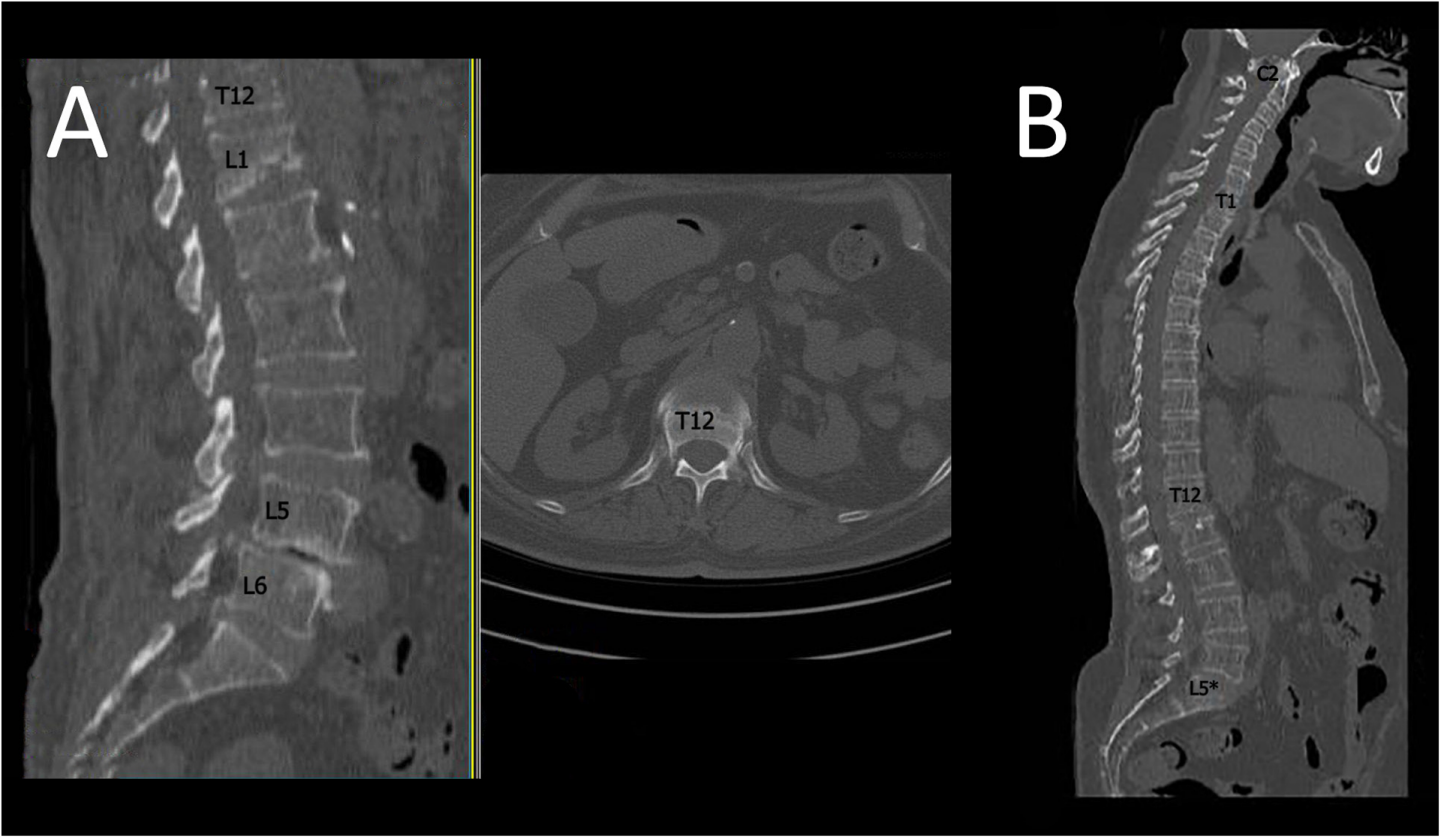
Spondylolisthesis patients with sacralization and lumbarization. **A**: Spondylolisthesis with lumbarization at L5-L6 (lumbarized S1) level; **B**: Spondylolisthesis with sacralization at L5*(sacralized L5)-S1 level

279 patients were initially identified and evaluated according to the inclusion criteria. The researchers contacted the identified patients and informed them about the study. The research objectives and the required information were clearly explained to all patients. After obtaining consent from patients, all patients underwent CT imaging at the expense of the research group and were subsequently evaluated based on their CT scans (Fig. 1). The medical records were then evaluated regarding age, sex, clinical signs and symptoms, presence or absence of LSTV, type of LSTV based on the Castellvi classification, and the level and grade of spondylolisthesis according to the Meyerding classification. Patients were then divided into No LSTV, Sacralization, and Lumbarization groups. In addition, signs and symptoms were categorized into four main classes, including (1) Low back pain (LBP) with disability assessed by the Oswestry Disability Index (ODI) [18]; (2) Lower limb paresthesia; (3) Motor deficit (i.e., presence of paresis or plegia); (4) Sphincter dysfunction.

### The Oswestry Disability Index (ODI) and Meyerding and Castellvi classifications

ODI is the most commonly used questionnaire for disability due to low back pain in a hospital setting. It is a self-administered questionnaire divided into ten sections designed to assess the limitations of various activities of daily living. Each section is scored on a 0–5 scale, with 5 representing the greatest disability. The index is calculated by dividing the summed score by the total possible score, which is then multiplied by 2 to obtain the index and expressed as a percentage. Therefore, the denominator is reduced by 5 for every question not answered. If a patient marks multiple statements in a question, the highest-scoring statement is recorded as a true indication of disability. Zero is equal to no disability; a score of 0–20 reflects minimal disability, 21–40 moderate disability, 41–60 severe disability, 61–80 crippled, and 81–100 bed-bound. The treatment type is decided based on the clinical signs and symptoms and the degree of the patient’s disability. Patients with the progression of signs and symptoms and/or significant disability because of pain (ODI > 40%) and/or failure to respond to at least 6 months of conservative therapy; are a candidate for surgery [19]. Indeed, all of the patients in our investigation met at least one of these conditions and underwent surgical treatment.

The Meyerding classification system is used to evaluate the degree of spondylolisthesis. It divides spondylolisthesis into five grades, including Grade I (0–25%), Grade II (26–50%), Grade III (51–75%), Grade IV (76–100%), and Grade V, also known as spondylolisthesis (greater than 100%) [20]. In general, grades I and II are generally considered low-grade slips, while Grades III, IV, and V are considered high-grade slips [21]. Using CT imaging, the grade percentage is determined by drawing a line through the posterior wall of the superior and inferior vertebral bodies and measuring the translation of the superior vertebral body as a percentage of the distance between the two lines.

The Castellvi classification is used for both sacralization and lumbarization states [22] using CT imaging, and it classifies as follows: type I (enlarged and dysplastic transverse process with a height of at least 19 mm); type II (pseudo-articulation of the transverse process and sacrum with incomplete lumbarization/sacralization and enlargement of the transverse process with pseudo arthrosis); type III (fusion of the transverse process with the sacrum, and the presence of a complete lumbarization or sacralization; and type IV (the combination of type IIa on one side and type IIIa on the contralateral side). In types I-III, terms a and b refer to unilateral and bilateral, respectively. In the present study, the sacralized L5 vertebra is symbolized as L5*. In addition, a lumbarized condition is recognized by observation of a non-complete fusion of S1 and S2 in CT imaging, indicating an additional articulated vertebra. To clarify this condition, in this study, the non-fused S1 vertebra is called L6, and the previous S2 segment is called nS1 as the new S1. Anatomically, the lumbar S1 is attached to the rest of the sacrum, similar to sacralized L5. Hence, Castellvi’s classification can also be used for a lumbarized state.

### Statistical analysis

Data were collected as a checklist in the SPSS software version 22 and analyzed at a significance level of < 0.05. Results were presented as frequency (percentage) or Mean ± SD. The means of the variables were compared using the independent T-test and Chi-Square (*X*^*2*^). Additionally, multiple logistic regression analysis was used to assess the significance of age and sex variations in study groups.

## Results

### Demographics and morphological spinal features between No LSTV, Sacralization, and Lumbarization groups

Out of 279 identified patients, 60 patients were excluded. The mean age of spondylolisthesis patients in the No LSTV, Sacralization, and Lumbarization groups was 57.6 ± 11, 56.3 ± 11.3, and 52.9 ± 9.7, respectively. Most patients in the No LSTV and Sacralization groups were female, while the Lumbarization group had an equal number of males and females. In this regard, significant variations in gender prevalence were found between the three groups (*P* < 0.05). More detailed information is provided in Table 1. The frequency of lumbarization and sacralization in spondylolisthesis patients was 4.5% and 26%, respectively. A significant difference was observed in the level of spondylolisthesis in study groups (*P* < 0.05). In the sacralization group, there was a 70.2% prevalence for L4-L5* spondylolisthesis, while the predominant level in the No LSTV and Lumbarization groups was the L5-S1 and L5-L6 (lumbarized S1) levels with a prevalence of 67.8% and 100%, respectively. Moreover, 51.2% of patients with L4-L5 spondylolisthesis, had sacralization. The listhesis grade had a trend toward significance among study groups (*P* = 0.053), with a significant difference observed for a lower percentage of grade I cases and a higher percentage of grade II cases in the Sacralization group compared to the No LSTV group (*P* = 0.009 and *P* = 0.020, respectively). Notably, the prevalence of higher grades of spondylolisthesis was higher in the Sacralization group than in the other groups, with 56.1% of the Sacralization patients exhibiting grade II or III listhesis (Table 1).

**Table 1:** Comparison of patients’ Demographics and Morphological Spinal Features between no LSTV, Sacralization, and Lumbarization groups

Variables	No LSTV (*n* = 152)	Sacralization (*n* = 57)	Lumbarization (*n* = 10)	P-value
Age (year), mean ± SD*	57.6 ± 11	56.3 ± 11.3	52.9 ± 9.7	0.362
M/F** (n)	66/86	14/43	5/5	0.034
% of female	56.6	75.4	50.0	
**Level of spondylolisthesis**^**a**^, **n (%)**				
L1-L2	0	0	0	0.001
L2-L3	2 (1.3)	0	0	
L3-L4	9(5.9)	7(12.3)	0	
L4-L5/L5*	38 (25)	40(70.2)	0	
L5/L5*-S1	103(67.8)	10(17.5)	0	
L5-L6	–	–	10(100)	
L6-nS1	–	–	0	
**Grade of listhesis, n (%)**				
I	101(66.5)	25(43.9)	6(60)	0.053
II	49(32.2)	30(52.6)	4 (40)	
III	2(1.3)	2(3.5)	0	
IV	0	0	0	
V	0	0	0	
**Castellvi classification**^**b**^, **n (%)**				
Ia	–	29(50.9)	0	0.001
Ib	–	23(40.4)	0	
IIa	–	3(5.3)	0	
IIb	–	0	5(50)	
IIIa	–	1(1.8)	0	
IIIb	–	1(1.8)	3 (30)	
IV	–	0	2 (20)	

**a**; The L5-L6 and L6-nS1 levels are only defined for the lumbarization group due to the presence of an additional lumbar vertebra which is actually the previous S1 vertebra defined in the normal anatomical state and is now called the L6 vertebra. The nS1 vertebra is the previous S2 segment in the normal anatomical state, but in the lumbarized state, it is the new S1. The L5* denotes the sacralized L5 vertebra in the sacralization group. **b**; The Castellvi classification implies that the LSTV states classify the anatomical connection varieties at the junction of the lumbar spine and sacrum; therefore, it is not defined for the no LSTV group. As expected, the difference in the Castellvi classification between sacralization and Lumbarization groups was significant, which reflects the natural difference in the development of spinal vertebrae in these two states. ^*^M: male, F: female, ^**^SD: standard deviation. P-value < 0.05 is statistically significant

Additionally, as shown in Table 2, all of the patients in the study had disabilities owing to low back pain, and all patients had ODI scores above 20. The presence of paresthesia and sphincter dysfunction was insignificant between the three groups. However, the frequency of paresis was significant among the study groups (*P* < 0.05), and 82.4% of sacralized patients had at least some degree of it. No case of plegia was observed in our study (Table 2).

**Table 2:** The frequency of each sign or symptom in No LSTV, Sacralization, and Lumbarization groups

Variables	No LSTV	Sacralization	Lumbarization	P-value
Pain, n (%) ^a^	152 (100)	57 (100)	10 (100)	–
Paresthesia, n (%)	149 (98)	57 (100)	10 (100)	0.798
Paresis/plegia ^b^, n (%)	78 (51.3)	47 (82.4)	5 (50)	0.001
Sphincter dysfunction, n (%)	13 (8.5)	8 (14)	0	0.430

**a**; All patients complained of Low Back Pain and ODI scores > 20. **b**; No case of plegia was observed among the patients. P-value < 0.05 is statistically significant

### Comparison of age and sex variations between the study groups

For each of the three groups, the age and gender variation were compared to the other two groups by multiple logistic regression analysis, as shown in Table 3. In each group, the cut age was 60, according to the mean age presented in Table 1. Besides, although variations in age were not significant in any of the study groups, the gender differences were significant in the No LSTV (OR: 1.937, 95% CI: 0.277–0.959) and Sacralization (OR: 0.417, 95% CI: 0.212–0.822) groups, unlike the Lumbarization group.

**Table 3:** Assessment of the significance of age and sex variations in No LSTV, Sacralization, and Lumbarization groups, by multiple logistic regression analysis

Variables	OR^*^	95% C.I.**	P-value
**Absence (No LSTV) group**			
Age (> 60 vs. ≤60)	1.428	0.385–1.272	0.241
Sex (male vs. female)	1.937	0.277–0.959	0.035
**Sacralization group**			
Age (> 60 vs. ≤60)	0.827	0.444–1.541	0.550
Sex (male vs. female)	0.417	0.212–0.822	0.010
**Lumbarization group**			
Age (> 60 vs. ≤60)	0.358	0.074–1.725	0.183
Sex (male vs. female)	1.613	0.453–5.745	0.457

* C.I.: confidence interval; OR: odds ratio; P-value < 0.05 is statistically significant

## Discussion

This study demonstrated that LSTV is commonly observed in patients with spondylolisthesis. So far, many studies have evaluated the relationship between the presence of LSTV, especially sacralization, in spondylolisthesis and its clinical features. While some studies confirm the association of LSTV with an increased risk of degenerative changes over the transitional vertebra [23–26], conflicting findings also exist. For instance, Kong et al. reported no difference in the degree of anterior slippage and disc degeneration between patients with and without L5 sacralization in a sample of patients with degenerative spondylolisthesis [27]. However, the sample size in their study was relatively small. Similarly, some others found no association between sacralization and spondylolisthesis [28]. In contrast, Benlidayi et al. pointed out that LSTV is linked with various structural changes (such as vertebral endplate/disc degeneration, spondylolisthesis, and disc protrusion) at the interspace immediately above the transitional segment [29]. Besides, some authors indicated that LSTV alters spinopelvic parameters, predisposing individuals to spondylolisthesis and degenerative disc disease [29]. Yao et al. evaluated the association between lumbar sacralization and the degree of vertebral slippage and disc degeneration in patients with L4 spondylolysis [30]. In their study, 36% of the patients with L4 spondylolysis had sacralization. Additionally, the authors found that vertebral slip and disc degeneration were significantly greater in the sacralization group than in the normal group. Therefore, they concluded that the increased stability between a sacralized L5 and the sacrum may predispose the L4-L5 segment to greater instability and disc degeneration in patients with L4 spondylolysis. In our study, similar results were observed, and higher grades of listhesis were found in patients with sacralization, with a 56.1% prevalence for grades 2 and 3. From a biomechanical perspective, both the L5*(sacralized L5) vertebra in sacralization and the L6 (lumbarized S1) vertebra in lumbarization groups are, as mentioned earlier, at least partially connected to the rest of the sacrum, and thus can be considered as part of the sacral bone. Accordingly, the last moveable lumbar vertebra in the sacralization state is the L4 vertebra, whereas, in the lumbarization state, it is the L6. Hence, the lumbosacral junction region, which is typically the most common site of spondylolisthesis, is at the L4-L5* and L5-L6 (lumbarized S1) levels in cases of sacralization and lumbarization, respectively. In the sacralization state, the number of moveable lumbar levels decreases, unlike the normal or lumbarized conditions. Consequently, the levels above the L4-L5* have to bear higher pressures. Hence, the sacralized L5 predisposes the cephalad segments to higher mobility and pressures, increasing the risk for higher grades of spondylolisthesis and disc degeneration. In this regard, in a similar study by Kim et al., the degree of anterior slippage was measured by Meyerding’s grading and the percentage of the Taillard method [31]. The authors demonstrated that sacralized patients with spondylolisthesis exhibited significantly higher L4 slip defects compared to both normal and lumbarized patients, whereas less slippage was observed at the L5 vertebra in comparison to the other two groups. In addition, they stated no significant difference was observed between the normal and lumbaraized groups. Therefore, they concluded that more aggressive treatment is recommended in patients with sacralization and L4 isthmic defects [31]. These findings are consistent with our results indicating a higher prevalence of L4-L5 listhesis in the sacralization group (70.2%). Moreover, in another study by Kong et al. [27], the incidence of L4-L5 listhesis was higher in sacralized L5 patients. However, contradictory to our study, they reported no significant differences in their four radiographic parameters (i.e., anterior slippage of L4 on L5, facet orientation of L4-L5, facet osteoarthritis of L4-L5, and disc degeneration of L4-L5) between the patients with and without sacralization of L5. Nevertheless, their study had several limitations, including a small sample size that precludes definitive conclusions, and uncertainty regarding whether the advanced arthritic changes of the facet joints and disc degeneration observed in degenerative spondylolisthesis are the cause or the result of the condition. In our observation, the incidence of L4-L5 listhesis in sacralized patients was higher compared to the other groups. In previous research, no significant difference was cited for age variation between normal and LSTV patients with spondylolisthesis [32]. However, there is controversy regarding the correlation between LSTV and gender [1, 33–35]. In this regard, Jancuska et al., in a review of symptomatic LSTV, stated that the prevalence of the male gender is significantly higher in the general population [1]. In contrast, a study by Dar et al. found that sacralization and spondylolisthesis were independent of gender compared to patients with normal lumbosacral anatomy [28]. In addition, in their investigation, no association between sacralization and spondylolisthesis was found, and hence, they indicated that sacralization should not be considered an etiology for the development of spondylolisthesis [28]. In this study, the presence or absence of LSTV in spondylolisthesis patients was age-independent, but significant gender variation with female predominance was seen in the sacralization group. It must be noted that we have designed a strict criterion to neutralize the effect of other potential risk factors and etiologies (e.g., spinal injury and major trauma, cancer, osteoporosis, etc.). In this regard, the main objective of the present study was to assess the exact correlation between LSTV and spondylolisthesis as far as possible. Generally, it is possible that morphological, habitual, and occupational differences between male and female populations in different regions lead to various gender-related findings in different studies. However, no significant gender differences were observed in the Lumbarization group, which may be influenced by the small number of these patients in the study and mandate further evaluation.

To date, there is no valid information about the prevalence of LSTV in the general population of Iran. However, LSTV is a common finding in the general population, with a prevalence of 5–30% reported by other researchers and a higher prevalence for sacralization than lumbarization [36, 37]. The frequency of sacralization and lumbarization in spondylolisthesis patients in our study was 26% and 4.5%, respectively. These observations suggest that although LSTV is common in spondylolisthesis patients, it is not more frequent than its frequency in the general population. In this study, the significant presence of motor deficit signs and symptoms was also observed in the sacralization group, which may correspond to the higher grades of spondylolisthesis and the probable co-existence of other non-diagnosed spinal and vertebral pathologies in sacralized patients. Although no remarkable investigation was performed on the clinical signs and symptoms in spondylolisthesis with LSTV, the significantly higher rate of motor deficit signs or symptoms in sacralization patients may be dedicated to other clinical effects of sacralization anomalies, which may be easily missed or hardly recognized. As mentioned earlier, Yao et al. stated that the presence of sacralization accelerates lumbar disc degeneration above the level of sacralized L5 [30]. In another study on 200 patients in China, the role of LSTV in the pathogenesis of lumbar disc herniation was evaluated, and it was noted that in patients with sacralization, a greater degree of disc degeneration and hernia, especially in higher segments, is compared to the normal population [38]. Besides, Hizal et al. found that LSTV is associated with a higher incidence of intervertebral osteochondrosis and Modic type 2 changes [39]. Some other authors also found that LSTV increases the risk of lumbar degenerative spinal stenosis [34]. Overall, symptoms may originate from instability and degeneration of the levels above, as well as nerve root compression. Since each of the above processes is treated differently, it requires precision in the type and location of the pathology [17, 40–42].

### Limitation

The current study had some limitations. Firstly, the number of spondylolisthesis patients with lumbarization was limited. Additionally, as the assessment of spinopelvic parameters was not the main objective of our study, relevant data concerning the study groups (No LSTV, Sacralization, and Lumbarization) in spondylolisthesis patients were not collected, which could have provided more information about our study groups. Furthermore, it’s important to note that our findings might vary in a larger population with diverse ethnic backgrounds. Therefore, further multicenter studies with a more diverse population are recommended.

## Conclusions

LSTV is frequently seen in spondylolisthesis patients. Sacralization, the most frequent type of LSTV in spondylolisthesis patients, possibly leads to an increased risk for higher grades of vertebral slip as well as higher rates of motor deficit signs and symptoms in spondylolisthesis patients, probably due to biomechanical changes and hypermobility of segments above the sacralized L5 vertebra. The presence of sacralization significantly increases the incidence of higher levels of spondylolisthesis, especially the L4-L5*(sacralized L5) level. There is no relationship between age and the presence of LSTV in spondylolisthesis.

## Data Availability

The datasets supporting the conclusions of this article are available from the corresponding author upon reasonable request.

## Abbreviations

LSTV: lumbosacral transitional vertebrae
LBP: Low Back Pain
ODI: Oswestry Disability Index
